# Obesity Prevalence Among Low-Income, Preschool-Aged Children — New York City and Los Angeles County, 2003–2011

**Published:** 2013-01-18

**Authors:** Jackson Sekhobo, Lynn Edmunds, Shannon Whaley, Maria Koleilat

**Affiliations:** New York State Dept of Health; Public Health Foundation Enterprises WIC Program, Los Angeles, California

Recent studies have reported evidence of a leveling (1) and decline in childhood obesity prevalence in New York (2,3) and California (4). However, some areas of the United States continue to experience increases in the prevalence of childhood obesity (5). To assess differences and changes over time in early childhood obesity in the two most populous cities in the United States, obesity prevalence among low-income, preschool-aged children enrolled in the Special Supplemental Nutrition Program for Women, Infants, and Children (WIC) in New York City (NYC) was compared with obesity prevalence among WIC-enrolled children in Los Angeles County (LAC) during 2003–2011. In NYC, from 2003 to 2011, obesity prevalence decreased among blacks, whites, and Hispanics, but increased among Asians. In LAC, obesity prevalence decreased among Asians and increased and then decreased among blacks and Hispanics from 2003 to 2011. Hispanic WIC-enrolled children had the greatest prevalence of obesity for all years in both areas. In 2011, the obesity prevalence among Hispanics in NYC was 19.1%, compared with 21.7% in LAC. Comparisons of obesity prevalence data among cities and states might suggest interventions and policies to help reverse childhood obesity increases in some populations.

NYC and LAC were chosen for study because they have the two largest populations of WIC participants and have different climates, unique built and policy environments, and readily available anthropometric data for children aged 3 or 4 years. In addition to pregnant or postpartum, breastfeeding women, WIC serves infants and children aged <5 years.* In both New York and California, height and weight measures of WIC participants are taken every 6 months by WIC staff members trained according to standard protocols, or measures are taken at physicians’ offices within 60 days of the WIC certification visit. A recent study of height and weight measures taken in WIC clinics demonstrated that they are both valid and reliable estimates of true height and weight (6). Height is recorded to the nearest one-quarter inch (6.35 mm) and weight to the nearest one-quarter pound (113 g). Date of birth and race/ethnicity for each child are reported to WIC staff members by the parent or caregiver. All of these data are entered into statewide information systems in New York or California; thus, identical measures are available in the two states. All data for this study were extracted from New York and California WIC administrative data systems for the period 2003–2011. For each calendar year, the most recent height and weight measures were obtained among children aged 3 or 4 years who were active WIC participants during the month of March; children could be measured at both age 3 and age 4 years, with their measurements included in the respective calculations by age level. For consistency, NYC WIC data followed the same inclusion criteria as LAC WIC.

Recorded measures of height and weight were converted to metric equivalents, and body mass index (BMI) was computed as weight in kilograms divided by height in meters squared. Obesity was defined as an age- and sex-specific BMI at or above the 95th reference percentile of the 2000 CDC growth charts for the United States.† Biologically implausible measurements were excluded from analysis. The prevalence of obesity by age, race/ethnicity, and geographic area was calculated for each year from 2003 to 2011. Race/ethnicity was categorized as follows: Hispanic, black, white, Asian and “other.”§ In this report, persons identified as Hispanic might be of any race. Persons identified as black, white, Asian, or other race are non-Hispanic. The five racial/ethnic categories are mutually exclusive.

The number of children aged 3 or 4 years participating in WIC in NYC each year ranged from 53,247 in 2003 to 67,428 in 2011. In LAC, the number of participating children ranged from 149,503 in 2003 to 147,292 in 2011. Among children enrolled in the NYC WIC, the Hispanic population increased from 44.0% of the total in 2003 to 46.4% in 2011, the white population increased from 12.5% to 13.9%, and the Asian population increased from 5.8% to 12.9%; the black population decreased from 28.2% of the total in 2003 to 23.9% in 2011, and the “other” group decreased from 9.5% to 2.9%. Among children enrolled in the LAC WIC, the Hispanic population increased from 82.6% of the total in 2003 to 85.5% in 2011, and the “other” group” increased from 0.4% to 2.1%. The black population decreased from 7.9% of the total in 2003 to 6.3% in 2011, the white population decreased from 4.9% to 2.8%, and the Asian population decreased from 4.1% to 3.3% (Table).

In 2003, obesity prevalence among WIC children aged 3 or 4 years was lower in LAC (16.3% and 17.2%, respectively) than in NYC (18.9% and 19.9%, respectively). However, by 2005, obesity prevalence in LAC had exceeded NYC for both age groups and continued to do so throughout the study period. From 2005 to 2009, obesity prevalences in NYC continued to decrease, whereas in LAC prevalences among children aged 3 years increased until 2008 and then decreased from 2009 to 2011, and prevalences among children aged 4 years increased until 2009 and then decreased (Figure 1).

With the exception of 2011 in LAC, the prevalence of obesity was higher among children aged 4 years than among children aged 3 years in both areas each year throughout the study period (Figure 1). From 2003 to 2009, the prevalence of obesity decreased among NYC WIC-enrolled children aged 3 or 4 years from 18.9% to 15.1% and from 19.9% to 17.2%, respectively. In contrast, from 2003 to 2009, the prevalence of obesity increased among LAC WIC-enrolled children aged 3 or 4 years from 16.3% to 21.0% and from 17.2% to 22.4%, respectively. From 2009 to 2011, obesity prevalence decreased from 21.0% to 20.5% among LAC WIC-enrolled children aged 3 years and from 22.4% to 20.3% among those aged 4 years and stayed relatively flat among NYC WIC-enrolled children (Figure 1).

Hispanics not only accounted for the largest proportion of WIC children in both cities, but also had the highest prevalence of obesity every year (Figure 2). In NYC, obesity prevalence among Hispanic children decreased from 24.0% in 2003 to 19.7% in 2009 and was 19.1% in 2011. Obesity prevalence among Hispanic children in LAC increased from 17.8% in 2003 to 23.0% in 2009 and then decreased to 21.7% in 2011. Among black WIC-enrolled children in NYC, obesity prevalence decreased from 17.7% in 2003 to 15.0% in 2006, then leveled off, whereas among white children, a gradual decline in obesity prevalence was observed, from 10.6% in 2003 to 8.9% in 2011. Among Asian children in NYC, a slight increase in obesity prevalence was observed, from 11.5% in 2003 to 11.9% in 2011 (Figure 2).

In LAC, among all racial/ethnic populations of WIC-enrolled children except Asians, an increase in obesity prevalence was observed from 2003 to 2008, with the increase continuing to 2009 among Hispanics, followed by a decrease to 2011 among Hispanics and blacks. Obesity prevalence decreased among Asians in LAC from 13.9% in 2004 to 11.3% in 2011.

## Editorial Note

Changes in obesity prevalence from 2003 to 2011 among low-income, preschool-aged children enrolled in WIC in NYC and LAC differed overall and by age and racial/ethnic population. In NYC, obesity prevalence decreased among all populations except Asians and blacks over the study period; among blacks, the prevalence decreased from 2003 to 2007 and then increased. In LAC, obesity prevalence decreased among Asians and increased and then decreased among blacks and Hispanics from 2003 to 2011. These patterns are consistent with national data indicating that increases in obesity prevalence among preschool-aged and school-aged children have leveled off (1) and with reports of declines in childhood obesity in New York (3) and California (4). The LAC patterns also are similar to those for fifth, seventh, and ninth graders who underwent California physical fitness testing in their schools. Data for that age group show an increase in obesity prevalence from 18.9% in 1999 to 23.3% in 2005, before decreasing to 22.4% in 2010 (Los Angeles County Department of Public Health, unpublished data, 2012).

The divergent changes and relatively higher prevalence observed among children enrolled in WIC in LAC are consistent with the epidemiology of childhood obesity in the United States described in the mid-2000s (5). Potential explanations for the differences observed between NYC and LAC might include sociodemographic differences in the populations enrolled in WIC, differential changes in the built environment, and differences in the timeframe and details of populationwide obesity prevention policies. Higher proportions of Hispanic children, among whom obesity is more prevalent (1), were enrolled in WIC in LAC than in NYC each year; differences in the racial/ethnic composition of children enrolled in WIC partially explain the relatively higher prevalence estimates in LAC than in NYC during the study period. In addition, the makeup of the Hispanic populations in NYC and LAC differ considerably, with large numbers of persons from the Caribbean in NYC and from Mexico and Central American in LAC (7). More research is needed to examine the reasons behind increased obesity risk among Hispanic children. The observed upward and downward shifts in obesity prevalence among Asian children in NYC and LAC, respectively, further suggest that the divergent changes observed in the two areas might be explained, in part, by changing demographics of their respective Asian sub-populations. According to the 2010 Census population estimates, the predominant Asian subpopulations in LAC are Chinese and Filipino, whereas the two largest Asian subpopulations in NYC are Chinese and Asian Indian (8).

With regard to the potential role of differences and changes in the built environment in the two areas, a greater probability of obesity has been found among children in neighborhoods with the most unfavorable social conditions, such as unsafe surroundings (e.g., poor housing and lack of access to sidewalks, parks, and recreation centers), than among children not facing such conditions (9). Recent analyses linking WIC early childhood obesity data with neighborhood environment data in LAC suggest that neighborhood food environments (e.g., fast food restaurants and convenience stores) might increase obesity risk, particularly among children in Spanish-speaking households (10). Thus, explorations of differences in the built environment might shed light on the divergent data in NYC and LAC.

What is already known on this topic?

After several decades of increasing prevalence of obesity among children, recent reports indicate that in some areas of the United States, including New York and California, the prevalence might be stabilizing or even decreasing.

What is added by this report?

This report compares obesity prevalence among children in New York City and Los Angeles County who were enrolled in the Special Supplemental Nutrition Program for Women, Infants, and Children. In New York City, the prevalence of obesity among children aged 3 or 4 years in this group decreased from 18.9% and 19.9%, respectively, in 2003 to 14.5% and 16.9%, respectively, in 2011. In Los Angeles County, obesity prevalence among children aged 3 or 4 years in this group increased from 16.3% and 17.2% in 2003 to 21.0% and 22.1%, respectively, in 2008, before decreasing from 21.0% and 22.4% in 2009 to 20.5% and 20.3%, respectively, in 2011. Obesity prevalence also differed by racial/ethnic population.

What are the implications for public health practice?

Obesity prevalence remains high among certain populations of children in both New York and Los Angeles. Interventions targeted to those populations with higher prevalence might be considered to help reduce the prevalence of obesity.

During the past decade, NYC implemented multiple interventions to address childhood obesity (2), and these efforts took place earlier in NYC than they did in LAC. Thus, it is possible that interventions and policies aimed at prevention of childhood obesity might have taken effect earlier in NYC than in LAC. As part of its *Eat Well, Play Hard* initiative for comprehensive childhood obesity prevention, since 2001 the New York state WIC program has been promoting consistent, positive messages related to age-appropriate physical activity, fruit and vegetable consumption, low-fat/nonfat milk consumption, breastfeeding, and TV viewing/screen time. California was involved in the *Fit WIC* initiative in a few regions around the state and began regional efforts to enhance WIC staff health and wellness in 2004, but did not begin a statewide nutrition education campaign that involved all of LAC until early 2009. This campaign focused on the upcoming changes to WIC food packages, and provided an enhanced wellness module for WIC staff members as well as focused training and education for staff members and participants on avoiding overfeeding and increasing consumption of whole grain, fruits and vegetables, and lower fat milk. The timing of these intervention efforts in each area appear to align well with decreases in the prevalence of childhood obesity observed in NYC in 2004 and LAC in 2010. However, more research is needed to assess the success of state and regional efforts focused on reducing childhood obesity in the past decade.

The findings in this report are subject to at least three limitations. First, the data represent only those participants in WIC and from whom height and weight measures were obtained during each year of the study. Second, information on the national origin of WIC participants was not available; therefore, it was not possible to assess whether changes in the proportions of racial/ethnic subpopulations might have contributed to changes in obesity prevalence in the two areas. Finally, this comparative analysis of childhood obesity prevalence in LAC and NYC constitutes an exploratory ecologic study because no simultaneous comparison of prevalence was made for a specific exposure during the study period.

Evidence of recent improvement in obesity prevalence among preschool-aged children enrolled in LAC WIC, which serves nearly three times as many children aged 3 and 4 years as NYC WIC, suggests that some areas with large numbers of WIC-enrolled children might need more time than others to complete the full adoption and implementation of policies and environmental strategies for obesity prevention. In addition, the difference in childhood obesity prevalence among WIC-enrolled preschool-aged children in NYC and LAC corroborates recent evidence of geographic variation in childhood obesity prevalence in the United States and lends support to the need for further investigations of the potential role of changes in demographics and environments, and the effects of populationwide obesity prevention policies.

## Figures and Tables

**FIGURE 1:**
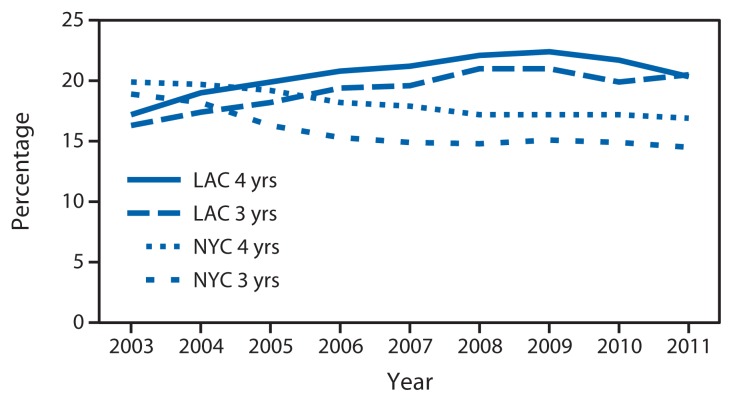
Prevalence of obesity* among children aged 3–4 years enrolled in the Special Supplemental Nutrition Program for Women, Infants, and Children, by age — New York City and Los Angeles County, 2003–2011 **Abbreviations:** NYC = New York City; LAC = Los Angeles County. * Obesity was defined as an age- and sex-specific body mass index at or above the 95th reference percentile of the 2000 CDC growth charts for the United States.

**FIGURE 2:**
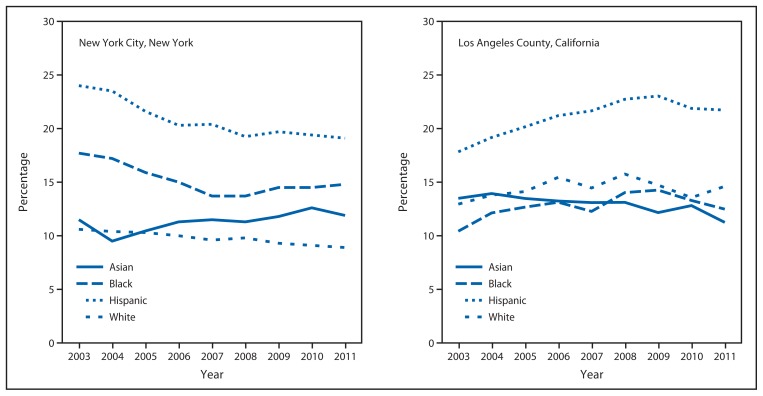
Prevalence of obesity* among children aged 3–4 years enrolled in the Special Supplemental Nutrition Program for Women, Infants, and Children, by race/ethnicity^†^ — New York City and Los Angeles County, 2003–2011 * Obesity was defined as an age- and sex-specific body mass index at or above the 95th reference percentile of the 2000 CDC growth charts for the United States. ^†^ Persons identified as Hispanic might be of any race. Persons identified as Asian, black, or white are non-Hispanic. The four racial/ethnic categories are mutually exclusive.

**TABLE:** Percentage of children aged 3–4 years enrolled in the Special Supplemental Nutrition Program for Women, Infants, and Children, by age and race/ethnicity — New York City and Los Angeles County, 2003–2011

	2003	2004	2005	2006	2007	2008	2009	2010	2011
Characteristic	%	%	%	%	%	%	%	%	%
**New York City**									
**Total no.**	**53,247**	**55,808**	**59,385**	**58,993**	**57,353**	**57,970**	**63,539**	**65,088**	**67,428**
**Age (yrs)**	**%**	**%**	**%**	**%**	**%**	**%**	**%**	**%**	**%**
3	56.3	56.6	55.5	55.4	55.4	55.3	55.3	55.7	55.1
4	43.7	43.4	44.5	44.6	44.6	44.7	44.7	44.3	44.9
**Race/Ethnicity** *									
Asian	5.8	6.1	6.8	8.1	9.0	9.8	10.9	11.7	12.9
Black	28.2	28.0	28.3	28.0	26.8	25.8	25.0	24.3	23.9
Hispanic	44.0	44.6	45.7	47.2	47.5	47.9	47.8	47.8	46.4
White	12.5	12.1	12.2	12.6	12.7	12.8	12.8	12.8	13.9
Other†	9.5	9.2	6.9	4.1	4.0	3.8	3.5	3.2	2.9
**Los Angeles County**									
**Total no.**	**149,503**	**148,377**	**144,171**	**139,863**	**133,646**	**137,148**	**137,714**	**142,878**	**147,292**
**Age (yrs)**	**%**	**%**	**%**	**%**	**%**	**%**	**%**	**%**	**%**
3	51.6	51.4	51.0	51.3	51.8	51.8	51.8	52.6	51.9
4	48.4	48.6	49.0	48.7	48.2	48.2	48.2	47.4	48.1
**Race/Ethnicity** *									
Asian	4.1	4.2	4.1	3.8	3.8	3.7	3.6	3.4	3.3
Black	7.9	7.6	7.3	6.7	6.6	6.6	6.7	6.4	6.3
Hispanic	82.6	83.0	83.6	85.6	85.6	85.5	85.5	85.7	85.5
White	4.9	4.9	4.7	3.1	3.1	3.0	3.0	2.9	2.8
Other	0.4§	0.4§	0.3§	0.9†	0.9†	1.2†	1.3†	1.7†	2.1†

* Persons identified as Hispanic might be of any race. Persons identified as white, black, Asian, or other race are non-Hispanic. The five racial/ethnic categories are mutually exclusive.

† Includes multirace, American Indian or Alaska Native, Native Hawaiian or Other Pacific Islander, and “refused.”

§ Includes American Indian or Alaska Native and “refused.”

## Footnotes

* Eligibility criteria for WIC include a family income ≤185% of the poverty income threshold, based on U.S. Poverty Income Guidelines, available at http://aspe.hhs.gov/poverty/12poverty.shtml. A person who participates or has family members who participate in certain other benefit programs, such as Medicaid or Aid to Families with Dependent Children/Temporary Assistance to Needy Families, automatically meets the income eligibility requirement.

† Available at http://www.cdc.gov/growthcharts.

§ For all years of NYC data and 2003–2005 LAC data, “other” included the subcategories multirace, American Indian or Alaska Native, Native Hawaiian or Other Pacific Islander, and “refused.” For 2006–2011 LAC data, “other” included American Indian or Alaska Native and “refused.”
